# Predictors of Progression in Intraplaque Hemorrhage Volume in Patients With Carotid Atherosclerosis: A Serial Magnetic Resonance Imaging Study

**DOI:** 10.3389/fneur.2022.815150

**Published:** 2022-07-15

**Authors:** Lu Mingming, Peng Peng, Zhang Lichen, Liu Shaohua, Yuan Fei, Zhang Hongtao, Liu Shitong, He Yao, Zhao Xihai, Cai Jianming

**Affiliations:** ^1^Department of Radiology, The Fifth Medical Center of PLA General Hospital, Beijing, China; ^2^Department of Radiology, Pingjin Hospital, Characteristic Medical Center of Chinese People's Armed Police Force, Tianjin, China; ^3^State Key Laboratory of Kidney Disease, Beijing Key Laboratory of Aging and Geriatrics, The Second Medical Center of PLA General Hospital, Institute of Geriatrics, Beijing, China; ^4^Center for Biomedical Imaging Research, Department of Biomedical Engineering, Tsinghua University School of Medicine, Beijing, China

**Keywords:** carotid plaque, atherosclerosis, progression, risk factors, magnetic resonance imaging

## Abstract

**Background and Purpose:**

This study aimed to investigate the arterial disease risk factors for the progression of intraplaque hemorrhage (IPH) in patients with carotid atherosclerosis using serial high-resolution magnetic resonance (MR) imaging.

**Methods:**

Consecutive symptomatic patients who had MRI evidence of intraplaque hemorrhage present in the ipsilateral carotid artery with respect to the side of the brain affected by stroke or TIA were recruited in the study. All the patients underwent follow-up MR imaging at least 6 months after baseline. The annual change in IPH and other carotid plaque morphology was calculated, and a tertile method was used to classify the plaques as progressed or not with respect to IPH volume using the software CASCADE. Logistic regression and receiver operating characteristic (ROC) curve were conducted to evaluate the risk factors for the progression of IPH.

**Results:**

A total of thirty-four symptomatic patients (mean age: 67.1 years, standard deviation [SD]: 9.8 years, 27 men) were eligible for the final analysis, and contralateral plaques containing IPH were seen in 11 of these patients (making 45 plaques with IPH in total). During mean 16.6-month (SD: 11.0 months) follow-up, the overall annual change in IPH volume in 45 plaques with IPH was mean −10.9 mm^3^ (SD: 49.1 mm^3^). Carotid plaques were significantly more likely to be classified in progressed IPH group if the patient was taking antiplatelet agent at baseline (OR: 9.76; 95%CI: 1.05 to 90.56; *p* = 0.045), had a baseline history of current or past smoking (OR: 9.28; 95%CI: 1.26 to 68.31; *p* = 0.029), or had a larger baseline carotid plaque-containing vessel wall volume (OR: 1.36 per 10 mm^3^; 95%CI: 1.02 to 1.81; *p* = 0.032) after adjustments for confounding factors. ROC analysis indicated that the combination of these three risk factors in the final model produced good discriminatory value for the progressed IPH group (area under the curve: 0.887).

**Conclusions:**

Taking an antiplatelet agent at baseline, a baseline history of current or past smoking and larger baseline carotid plaque-containing vessel wall volume were independently predictive of plaques being in the progressed IPH group. Our findings indicate that awareness and management of such risk factors may reduce the risk of intraplaque hemorrhage progression.

## Introduction

Intraplaque hemorrhage (IPH), as one of the key characteristics of vulnerable plaques in carotid arteries, has been demonstrated to have a significant association with future ischemic events (1–6). Hennings et al. (1) investigated the relationship between the histologically determined atherosclerotic plaque composition and the occurrence of future vascular events, and they found that patients whose excised carotid plaque revealed plaque hemorrhage demonstrated an increased risk of cerebrovascular ischemic events. Furthermore, Kurosaki et al. (3) also reported that patients with carotid artery lesions and intraplaque hemorrhage tended to be at higher risk of subsequent ipsilateral ischemic events.

High-resolution multi-contrast magnetic resonance imaging (MRI) has been demonstrated to be capable of detecting IPH in carotid arteries validated by histology. Cai et al. (7) analyzed the pathological specimens obtained from patients with carotid atherosclerosis and found that the sensitivity and specificity of MRI for identifying plaques with IPH were 82 and 91%, respectively.

Furthermore, MRI exhibits good reproducibility in evaluating the changing characteristics of IPH in carotid artery (8, 9). Li et al. (8) evaluated the scan–rescan reproducibility of plaque compositions and found that hemorrhages were repeatedly detected on both scans in the same subjects with perfect agreement (kappa = 1). Similarly, our results showed that the intraclass correlation coefficient (ICC) for the intraobserver agreement in measurements of IPH volume in carotid artery was 0.86.

Previous studies have investigated the risk factors for the presence of IPH, which showed that increasing patient's age, male sex, increase in degree of stenosis, or elevated lipid levels indicated a higher likelihood for the presence of intraplaque hemorrhage (9, 10). However, there are few studies that examine the risk factors for the progression of IPH, although the change in IPH has been investigated by previous studies using serial MR examinations (11, 12). Further studies are warranted to identify the arterial disease risk factors for the progression of IPH, because risk factors for progression may be different to risk factors for the presence of IPH. Furthermore, intervention may reduce the risk of progression once evidence of IPH is detected and prevention of IPH progression may reduce the risk of arterial disease complications, including stroke.

Therefore, this study aimed to investigate the changing characteristics of carotid IPH and the arterial disease risk factors that are related to the progression in IPH in patients with carotid atherosclerosis using serial high-resolution magnetic resonance (MR) imaging.

## Materials and Methods

### Study Sample

Consecutive patients with a recent (<3 months) cerebral ischemic stroke or transient ischemic attack (TIA) who exhibited atherosclerotic wall thickening with intraplaque hemorrhage in ipsilateral carotid arteries determined by MR vessel wall imaging were recruited from a single institution. All the recruited patients underwent a follow-up MR vessel wall imaging with an interval of at least 6 months from baseline. The exclusion criteria were as follows: (1) patients with other cerebral disorders such as brain tumor that may receive antitumor therapy and have potential influence on the progression of IPH; (13) (2) pregnancy; (3) contraindication to MR examination; (4) insufficient longitudinal coverage of carotid plaque on MR images; and (5) patients who had an ipsilateral carotid stent or carotid endarterectomy. Age, gender, body mass index (BMI), hypertension, hyperlipidemia, diabetes, coronary artery heart disease, current smoking or previous smoking history, and current medications (including the use of statin and antiplatelet agents) were collected and ascertained at the time of baseline MR imaging.

Hypertension was considered present when the systolic blood pressure exceeded 140 mmHg or the diastolic blood pressure exceeded 90 mmHg. Hyperlipidemia was diagnosed when the low-density lipoprotein exceeded 1.58 mmol/L, total cholesterol exceeded 2.26 mmol/L, or triglycerides exceeded 1.69 mmol/L. Diabetes mellitus was considered present if the fasting blood sugar level was more than 126 mg/dL, the 2-h oral glucose tolerance test result was more than 200 mg/dL, or the hemoglobin A1c was at least 6.5%. Participants were defined as smokers if they had ever smoked or were current smokers. TIA was defined as the rapidly developing signs of a neurological deficit or loss of vision, lasting <24 h with no apparent cause other than that of vascular origin. Ischemic stroke was defined as the rapidly developing clinical signs of a neurological deficit, lasting at least 24 h with no apparent cause other than that of vascular origin and without evidence of an intracranial hemorrhage on CT/MR images. The study protocol was approved by institutional review board and all participants provided the written informed consent.

### Vessel Wall MR Image Acquisition

All MR imaging was conducted with the same 3.0-T MR unit (SignaHDx, GE Medical System, Milwaukee, WI, USA) at both time points with a 4-channel dedicated phase-arrayed surface coil. The vessel wall MR protocol was acquired with the following parameters: (1) 3D time of flight (TOF): 3D gradient echo (GRE), repetition time (TR)/echo time (TE) = 29 /2.1 ms, flip angle = 20°, field of view (FOV) of 140 mm × 140 mm, matrix size of 256 × 256, and slice thickness of 2 mm; (2) T1W: fast spin echo (FSE), TR/TE = 800 /7.5 ms, flip angle = 90°, FOV of 140 mm × 140 mm, matrix size of 256 × 256, and slice thickness of 2 mm; and (3) T2W: FSE, TR/TE = 3000 /57 ms, flip angle = 90°, FOV of 140 mm × 140 mm, matrix size of 256 × 256, and slice thickness of 2 mm. The MR scan was centered to the bifurcation of the index carotid artery, which is defined as the arteries that were associated with ipsilateral cerebral ischemic stroke or TIA and plaques from the contralateral carotid artery were also evaluated. The baseline and follow-up MR images were acquired using the same vessel wall imaging protocol.

### Vessel Wall MR Image Analysis

A number of two trained radiologists with more than 2 years of experience in plaque imaging were blinded to the patients' clinical information and the imaging time point and analyzed the MR images using custom-designed software CASCADE (Vascular Imaging Lab, University of Washington) (14). A 4-point scale (1 = poor, 2 = marginal, 3 = good, and 4 = excellent) was utilized to assess the image quality per slice, and patients who had any images with quality score <2 were excluded from the study.

**Figure 1:**
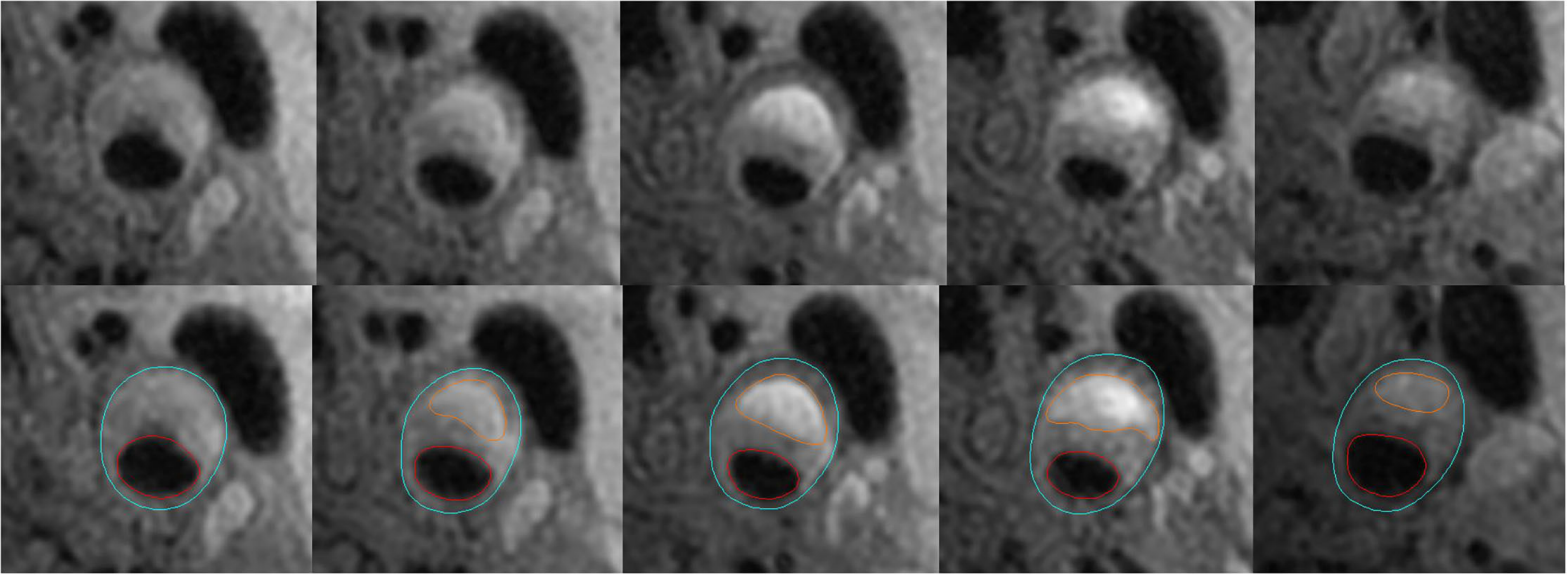
Example for measurements of carotid plaque-containing vessel wall volume and IPH volume. The figure shows that the lumen in red and outer wall boundary in blue of the carotid artery are identified and outlined on T1-weighted images. Carotid plaque-containing vessel wall volume is calculated by multiplying the slice thickness (2 mm) by the sum of the areas between the red and blue circles in slices with a vessel wall thickness of at least 2 mm. IPH volume is calculated by multiplying the slice thickness (2 mm) by the sum of the areas circled in orange color.

Carotid plaque was defined as a wall thickening ≥2 mm in carotid artery and the measuring of the wall was defined as the distance between the lumen boundary and outer wall boundary which was illustrated in Figure 1. The presence or absence of plaque compositions, such as IPH and calcification, was identified using the published criteria (15). The presence or absence of fibrous cap rupture (FCR) was also determined (15). The IPH was defined as hyperintense compared with adjacent muscle on both T1W and 3D TOF images. The FCR was defined as the irregular surface with the absence of the dark band between the bright lumen and the gray plaque core on 3D TOF images. Plaque calcification was defined as hypo-intense on all image sequences.

Because it is hard to identify the boundary of an atherosclerotic plaque using MRI, the carotid plaque-containing vessel wall volume was used instead of the carotid plaque volume in this study (2, 11). In this study, the volume of vessel wall containing the plaque was calculated using the sum of total vessel wall area in slices with a wall thickness of at least 2 mm multiplied by slice thickness (2 mm) (Figure 1). The volume of IPH was calculated as the sum of corresponding IPH area in each slice multiplied by slice thickness (2 mm) (Figure 1). The change in IPH volume was obtained by the IPH volume measured at the second MR examination minus that at the first MR examination using the software CASCADE. The minimum discernible change in volume using the software CASCADE is 0.1 mm^3^. The progression of IPH volume included an increased volume of hemorrhage in the same intraplaque location seen at baseline and any new areas of hemorrhage seen in the same plaque. The luminal stenosis of carotid arteries was measured on maximum intensity projection images reconstructed from the 3D TOF sequence and calculated with the formula of 1-(narrow lumen diameter/reference lumen diameter) ^*^100%. The reference site was defined using a vessel segment with a normal appearance distal to the stenotic segment (16).

In addition to measuring the progression of IPH volume, we also evaluated the progression using visual inspection. The minimum discernible visible change in IPH volume with respect to progression or regression was defined as a change >10 mm3. Carotid plaques were stratified into tertiles (2, 16) according to the annual change in IPH volume which was measured and calculated using the software CASCADE. The highest tertile was defined as the progressed IPH group, and the other two tertiles were defined as the non-progressed IPH group. Tertile method was used in this study, because it is inappropriate to use the absolute value “0” of annual change in IPH volume as a critical threshold to separate the progressed IPH group from non-progressed IPH group, because small changes in measurements might be caused by a measurement error and not a real progression or regression. Plaques with the minimal discernible visible change in IPH volume were all distributed in the highest tertile. The progressed IPH group was referred to the highest tertile of change in IPH volume, and the grouping and correlation analysis were performed based on the tertile method.

### Statistical Analysis

Continuous variables were summarized as mean ± standard deviation (SD) and categorical variables were presented as percentages. The annual change (mean change per year) of plaque-containing vessel wall volume, IPH volume, maximum carotid wall thickness, and stenosis of carotid artery were calculated. The clinical characteristics and carotid plaque measurements were compared between the progressed IPH and non-progressed IPH groups using independent Student *t*-test, Mann–Whitney U test or chi-square test. Pearson correlation analysis was conducted to determine the relationship between annual change in carotid plaques containing vessel wall volume and the progressed IPH group.

Univariate and multivariate logistic regressions were used to evaluate the arterial disease risk factors related to the progressed IPH group. Age, gender, body mass index (BMI), hypertension, hyperlipidemia, diabetes, coronary artery heart disease, current smoking or previous smoking history, current medications, and baseline characteristics of carotid plaques (vessel wall volume, stenosis, maximum wall thickness, IPH volume, presence of calcification, and the presence of FCR) were used in the univariant analysis to test the association between arterial disease risk factors and the progressed IPH group. Age, gender, and BMI and variables in the univariate analysis with *p* < 0.05 were adjusted in the logistic analysis. Variables in the univariate analysis with *p* ≥ 0.05 were not considered in the multivariate logistic regression analysis. The intraobserver and interobserver agreement in measurements of carotid plaque-containing vessel wall volume, degree of stenosis, maximum wall thickness, and IPH volume in carotid artery and identification of plaque compositions were evaluated by intraclass correlation coefficient (ICC) and Cohen's kappa test, respectively. The two-sided *p* < 0.05 was considered as statistically significant and all statistical analyses were performed with SPSS, version 22.0 (IBM, Chicago, IL).

## Results

In total, 39 patients with carotid plaques ipsilateral to the symptomatic brain region/eye and containing IPH were enrolled in the study from January 2005 to November 2011. Of these, 5 patients were excluded because of poor image quality (n = 2), insufficient longitudinal coverage (n = 3), and other cerebral disorders such as brain tumor (n = 0). Of the remaining 34 patients (mean age: 67.1 ± 9.8 years old) who were finally included for statistical analysis, 27 (79.4%) were men, 24 (70.4%) had hypertension, 19 (55.9%) had hyperlipidemia, 9 (26.5%) had diabetes, and 14 (41.2%) had a baseline history of current or past smoking. The detailed clinical characteristics of the sample at baseline are shown in Table 1. The mean time interval between the two MRI scans was 16.6 months (standard deviation [SD]: 11.0 months) (range: 6−36 months).

**Table 1:** Subject characteristics at baseline.

**Clinical characteristics**	**Patients with IPH**
	**(*n* = 34)**
Gender, male (%)	27 (79.4)
Age (years)	67.1 ± 9.8
BMI (kg/m^2^)	23.7 ± 2.7
Hypertension (%)	24 (70.4)
Hyperlipidemia (%)	19 (55.9)
Diabetes (%)	9 (26.5)
LDL cholesterol (mmol/L)	2.6 ± 1.2
HDL cholesterol (mmol/L)	1.2 ± 0.8
Total cholesterol (mmol/L)	4.3 ± 1.2
Triglyceride (mmol/L)	1.6 ± 1.0
Antiplatelet agent (%)	21 (61.8)
Use of statin (%)	16 (47.1)
Smoking (%)	14 (41.2)
Coronary heart disease (%)	13 (38.2)
Interval time of follow up (months)	16.6 ± 11.0

*BMI, body mass index; LDL, low-density lipoprotein; and HDL, high-density lipoprotein. Antiplatelet agent, taking an antiplatelet agent at baseline; smoking, a baseline history of current or past smoking*.

### The Baseline Characteristics of Carotid Plaques

Among the 34 symptomatic patients included in this study, all had baseline MRI evidence of ipsilateral carotid intraplaque hemorrhage and 29 had bilateral carotid plaques. The mean degree of stenosis in the 34 ipsilateral carotid plaques with respect to previous stroke or TIA and for the plaques in the contralateral carotid arteries was 55.2% (SD: 11.8%, range: 32.1–78.8%) and 45.5% (SD: 12.2%, range: 33.3–66.4%), respectively. Among the 63 total carotid plaques detected at baseline, 45 had MRI evidence of intraplaque hemorrhage. Of the 45 plaques with IPH, 37 (82.2%) had calcification and 14 (31.1%) had fibrous cap rupture. With respect to 45 plaques with IPH, the average value of carotid plaque-containing vessel wall volume (mean 955.6 mm^3^, SD: 391.7 mm^3^, range: 461.7 mm^3^-2,136.9 mm^3^), degree of stenosis (mean 52.8%, SD: 12.5%, range: 32.1–78.7%), maximum wall thickness (mean 4.9 mm, SD: 1.3 mm, range: 2.5–7.7 mm), and IPH volume (mean 220.6 mm^3^, SD: 91.9 mm^3^, range: 77.1−534.2 mm^3^) at baseline was obtained.

### The Changing Characteristics of Carotid Plaques

During mean 16.6-month (SD: 11.0 months) follow-up, with respect to 45 plaques with IPH, the mean annual change in carotid plaque-containing vessel wall volume (mean 19.7 mm^3^, SD: 63.2 mm^3^, range:−103.2−162.3 mm^3^), degree of stenosis (mean 1.2%, SD: 7.6%, range:−27.1–26.9%), and maximum wall thickness (mean 0.5 mm, SD: 1.3 mm, range:−2.7−3.4 mm) were obtained. The overall annual change in intraplaque hemorrhage volume which was measured using the software CASCADE in these 45 plaques during the average follow-up of 16.6 months was that of regression,−10.9 mm3 (SD: 49.1 mm^3^, range:−138.2−135.2 mm^3^).

**Figure 2:**
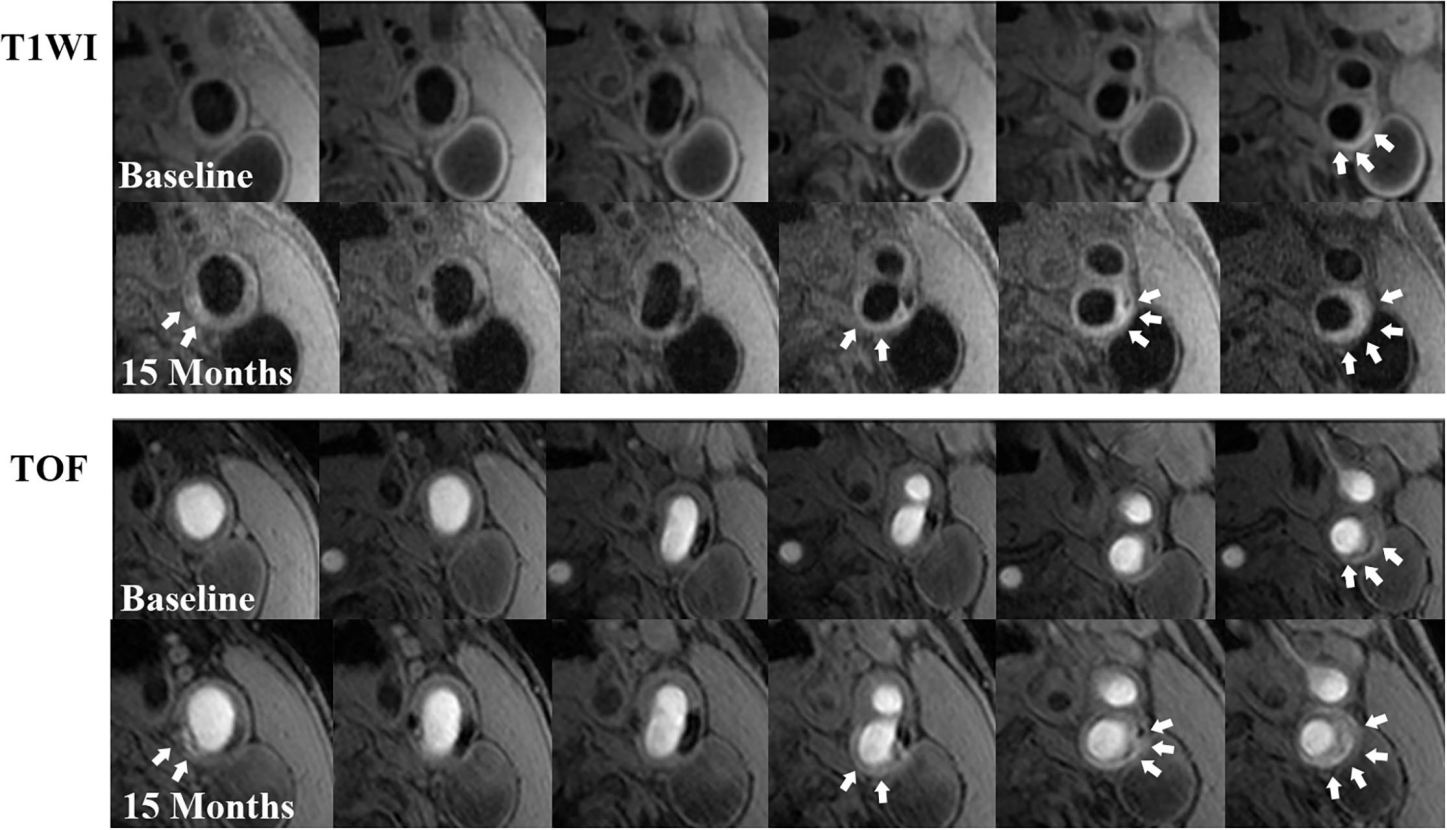
Examples of IPH progression during a 15-month follow-up. There is a remarkable progression in IPH volume (white arrows) at left carotid bifurcation with atherosclerosis on T1W (above 2 rows) and TOF (below 2 rows) images from the baseline to the follow-up. In addition, new IPHs (white arrows) could be observed at other locations of the plaque on follow-up MR images compared with those at baseline.

**Figure 3:**
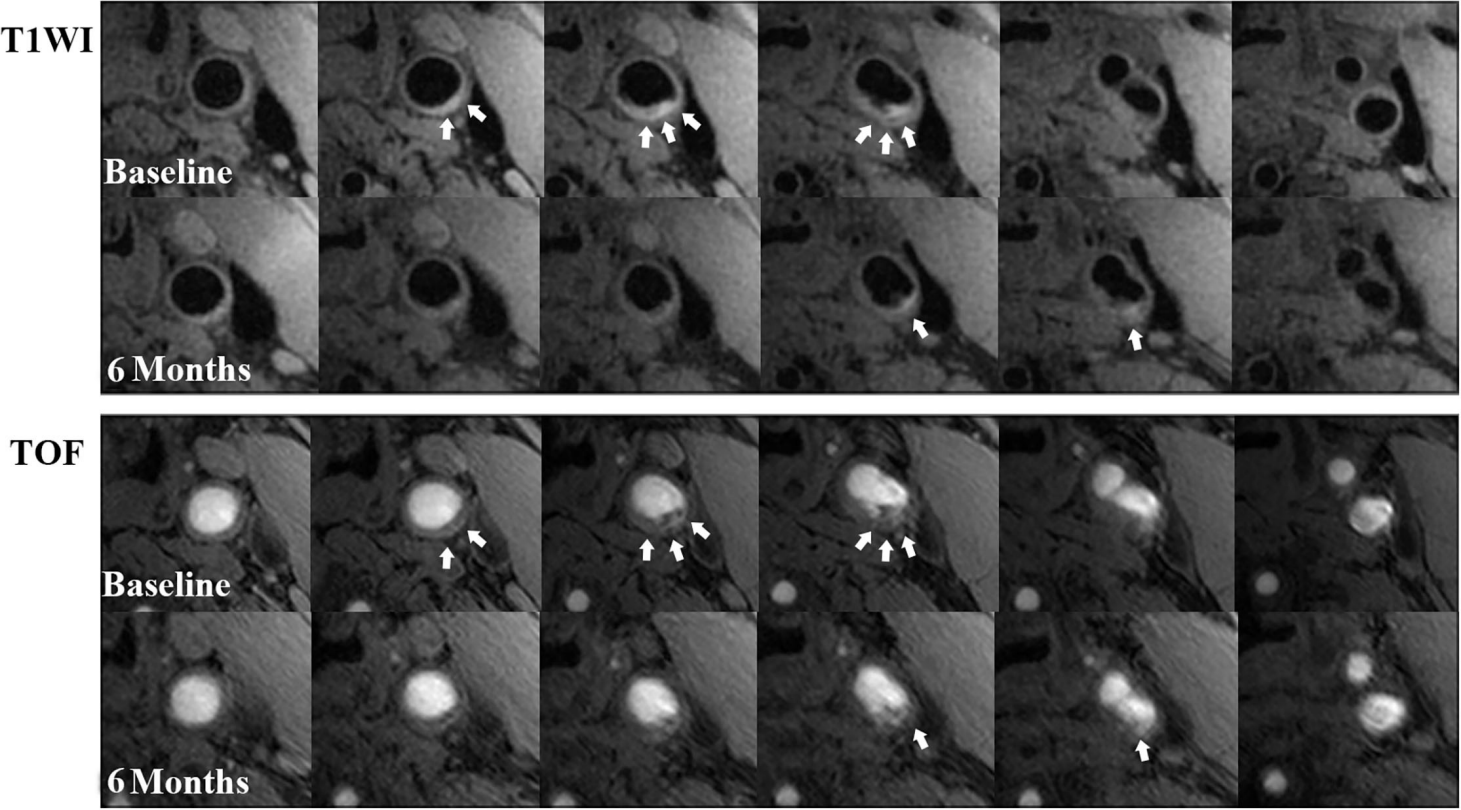
Examples of IPH regression during a 6-month follow-up. There is a remarkable regression in IPH volume (white arrows) at left carotid common artery with atherosclerosis on T1W (above 2 rows) and TOF (below 2 rows) images from the baseline to the follow-up. On some slices of the follow-up MR images, the hyperintense signal (IPH) disappeared compared with those at baseline.

The annual change in IPH volume in the 15 plaques in the progressed IPH group was mean 27.1 mm^3^ (SD: 36.5 mm^3^, range: 2.5−135.2 mm^3^) (Figure 2). The annual change in IPH volume in the 30 plaques in the non-progressed IPH group was mean−30.0 mm^3^ (SD: 43.3 mm^3^, range:−138.2–1.78 mm^3^) (Figure 3). No differences in the prevalence of IPH progression between the carotid plaques with follow-up interval of 6–18 months and those with 18–36 months (27.8 vs. 37.0%, *p* = 0.748) were found. None exhibited complete loss of hyperintensity of IPH during the follow-up compared to baseline. Carotid plaques in the progressed IPH group had greater annual change in plaque-containing vessel wall volume (mean 50.3 mm^3^, SD: 57.4 mm^3^, range:−37.7–147.9 mm^3^) compared to those in the non-progressed IPH group (*p* = 0.020). The annual change in plaque-containing vessel wall volume in the non-progressed IPH group was mean 4.5 mm^3^ (SD: 61.3 mm^3^, range:−103.2–162.3 mm^3^). The annual change in IPH volume was positively correlated with the change in plaque-containing vessel wall volume (*r* = 0.491, *p* = 0.001).

With respect to progression of IPH volume, as mentioned in Methods, this included expansion of areas of intraplaque hemorrhage seen at baseline and areas of new intraplaque hemorrhage within the same plaque on the second MRI. Of the 45 plaques with IPH, 22.2% (10/45) had minimal discernible visible change in IPH volume and this included 11.1% (5/45) who had new areas of IPH at other locations in the same carotid plaque.

### Association Between Arterial Disease Risk Factors and the Progressed IPH Group

With respect to the 45 plaques with IPH, those plaques were significantly more likely to be in the progressed IPH group compared to the non-progressed IPH group if the patient had been on an antiplatelet agent at baseline (86.7 vs. 53.3%, *p* = 0.046), had a baseline history of current or past smoking (73.3 vs. 30.0%, *p* = 0.010), or had larger baseline carotid plaque-containing vessel wall volume (mean 1154.3 mm^3^, SD: 473.1 vs. mean 856.2 mm^3^, SD: 306.7 mm^3^, *p* = 0.014) (Table 2). Each of these associations (antiplatelet agent, history of smoking, and larger baseline plaque-containing vessel wall volume) was each separate predictor of plaques being in progressed IPH group. There were no other significant differences in clinical parameters and measurements of carotid arteries at baseline between the two groups (all *p* > 0.05).

**Table 2:** Comparison of clinical and morphological characteristics between plaques in the progressed and non-progressed IPH groups.

	**Mean ±SD or** ***n*** **(%)**		***p***
	**Plaques in non-progressed IPH group**	**Plaques in progressed IPH group**	
	**(*n* = 30)**	**(*n* = 15)**	
**Clinical characteristics at baseline**			
Gender, male	25 (83.3)	11 (73.3)	0.454
Age	66.6 ± 9.9	72.2 ± 7.9	0.063
BMI	23.6 ± 2.5	23.7 ± 3.1	0.971
Hypertension	21 (70.0)	12 (80.0)	0.722
Hyperlipidemia	20 (66.7)	6 (40.0)	0.116
Diabetes	7 (23.3)	5 (33.3)	0.496
Antiplatelet agent	16 (53.3)	13 (86.7)	0.046
Use of statin	16 (53.3)	7 (46.7)	0.758
Smoking	9 (30.0)	11 (73.3)	0.010
Coronary heart disease	10 (33.3)	6 (40.0)	0.746
**Baseline characteristics of carotid plaques**			
Vessel wall volume, cm^3^	856.2 ± 306.7	1154.3 ± 473.1	0.014
Stenosis, %	51.9 ± 13.1	54.7 ± 11.4	0.485
Maximum wall thickness, mm	4.7 ± 1.4	5.2 ± 1.1	0.264
IPH volume, mm^3^	233.8 ± 97.1	194.1 ± 76.7	0.174
Presence of calcification	24 (80.0)	13 (86.7)	0.699
Presence of FCR	8 (26.7)	6 (40.0)	0.497
**The annual change in carotid plaque characteristics**			
Vessel wall volume, mm^3^	4.5 ± 61.3	50.3 ± 57.4	0.020
Stenosis, %	0.6 ± 8.6	2.1 ± 5.5	0.550
Maximum wall thickness, mm	0.3 ± 0.8	0.8 ± 0.9	0.163
IPH volume, mm^3^	−30.0 ± 43.3	27.1 ± 36.5	<0.001^‡^

*Vessel wall volume, carotid plaque-containing vessel wall volume; IPH, intraplaque hemorrhage; BMI, body mass index; FCR, fibrous cap rupture. ^‡^The result is only because of the patients' stratification. Antiplatelet agent, taking an antiplatelet agent at baseline; smoking, a baseline history of current or past smoking*.

Logistic regression analysis showed that taking an antiplatelet agent at baseline (OR: 5.69; 95%CI: 1.09 to 29.69; *p* = 0.039), a baseline history of current or past smoking (OR: 6.42; 95%CI: 1.61 to 25.64; *p* = 0.009) and larger baseline carotid plaque-containing vessel wall volume (OR: 1.23 per 10 mm^3^; 95%CI: 1.03 to 1.47; *p* = 0.025) were predictive of plaques being progressed IPH group (Table 3). Multivariate logistic regression analysis revealed that the associations of taking an antiplatelet agent at baseline (OR: 9.76; 95%CI: 1.05 to 90.56; *p* = 0.045), a baseline history of current or past smoking (OR: 9.28; 95%CI: 1.26 to 68.31; *p* = 0.029), and larger baseline carotid plaque-containing vessel wall volume (OR: 1.36 per 10 mm^3^; 95%CI: 1.02 to 1.81; *p* = 0.032) with the progressed IPH group remained statistically significant after adjustments for confounding factors (refer to Table 3). No significant associations could be found between other carotid plaque characteristics and the progressed IPH group before and after adjustments for above confounding factors (all *p* ≥ 0.05).

**Table 3:** Logistic regression models of risk factors for the progressed IPH group.

	**Progressed IPH group**					
	**Univariate regression**			**Multivariate regression**		
	**OR**	**95%CI**	***p***	**OR**	**95%CI**	***P***
**Clinical characteristics at baseline**						
Gender, male	1.82	0.41–8.10	0.433	0.99	0.04–25.19	0.996
Age	1.07	0.99–1.17	0.072	1.09	0.94–1.26	0.267
BMI	1.00	0.80–1.27	0.970	1.00	0.67–1.50	0.995
Hypertension	1.71	0.39–7.58	0.477	0.50	0.04–5.98	0.581
Hyperlipidemia	0.33	0.09–1.20	0.093	0.38	0.05–3.16	0.370
Diabetes	1.64	0.42–6.45	0.477	0.22	0.01–3.64	0.288
Antiplatelet agent	5.69	1.09–29.69	0.039	9.76	1.05–90.56	0.045
Use of statin	0.77	0.22–2.65	0.674	0.04	0.001–1.55	0.084
Smoking	6.42	1.61–25.64	0.009	9.28	1.26–68.31	0.029
Coronary heart disease	1.33	0.37–4.81	0.660	0.23	0.02–2.58	0.234
**Characteristics of carotid plaques at baseline**						
Vessel wall volume, mm^3^*	1.23	1.03–1.47	0.025	1.36	1.02–1.81	0.032
Stenosis, %^‡^	1.20	0.72–1.99	0.475	1.52	0.67–3.48	0.320
Maximum wall thickness, mm§	1.32	0.81–2.16	0.259	2.11	0.82–5.43	0.121
Baseline IPH volume, mm^3^*	0.57	0.25–1.29	0.178	0.48	0.14–1.65	0.243
Presence of FCR	1.83	0.49–6.81	0.365	4.37	0.41–46.31	0.220
Presence of calcification	1.63	0.29–9.23	0.584	3.62	0.23–57.19	0.361

Vessel wall volume, carotid plaque-containing vessel wall volume; IPH, intraplaque hemorrhage; BMI, body mass index; FCR, fibrous cap rupture; increment, ^*^10 mm^3^; ^‡^, 1%; §, 1 mm. Multivariate regression was adjusted for baseline IPH volume; age; gender; BMI; and all other covariates in the univariate analysis with p < 0.05 (antiplatelet agent; smoking; and plaque-containing vessel wall volume), when applicable. Antiplatelet agent, taking an antiplatelet agent at baseline; smoking, a baseline history of current or past smoking.

**Figure 4:**
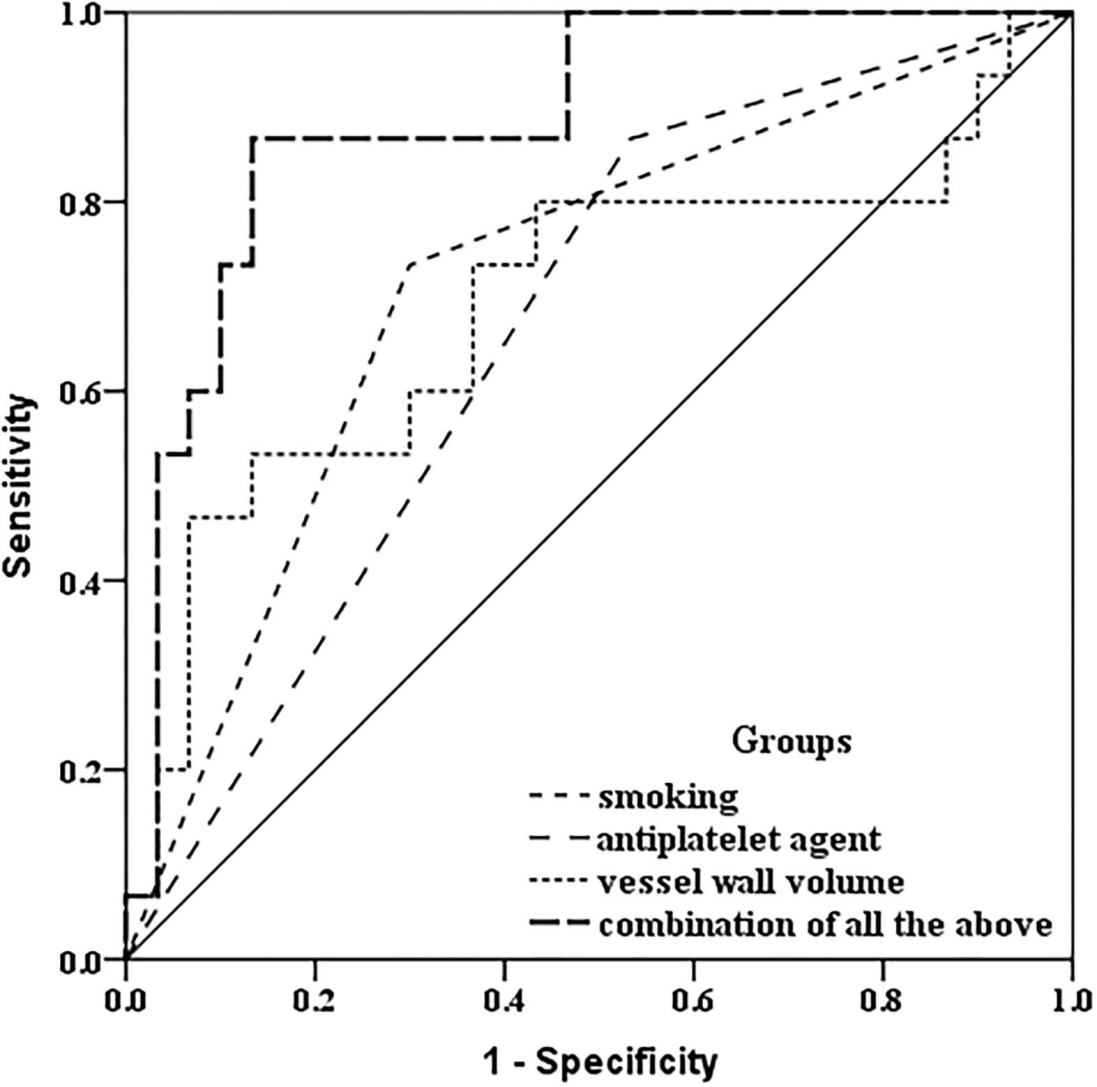
Receiver operating characteristic comparison analysis. In discriminating the progressed IPH group, receiver operator curve analysis indicated that combined with a baseline history of current or past smoking, taking an antiplatelet agent at baseline, and larger baseline carotid plaque-containing vessel wall volume, there was incremental improvement in the area under the curve (AUC: 0.887) compared with a baseline history of current or past smoking only (AUC: 0.717), taking an antiplatelet agent at baseline only (AUC: 0.667), and larger baseline carotid plaque-containing vessel wall volume (AUC: 0.691) only.

In discriminating the progressed IPH group, receiver operator curve analysis indicated the combination of taking an antiplatelet agent at baseline, a baseline history of current or past smoking, and larger baseline carotid plaque-containing vessel wall volume, there was incremental improvement in the area under the curve (AUC: 0.887) compared with taking an antiplatelet agent at baseline (AUC: 0.667), a baseline history of current or past smoking (AUC: 0.717), or larger baseline carotid plaque-containing vessel wall volume alone (AUC: 0.691) (Figure 4).

### Reproducibility

In this study, the intraclass correlation coefficients (ICCs) for the intraobserver agreement in measurements of carotid plaque-containing vessel wall volume, degree of stenosis, maximum wall thickness, and IPH volume in carotid artery were 0.92, 0.84, 0.85, and 0.86, respectively. For the interobserver agreement, the ICCs were 0.90, 0.81, 0.87, and 0.88, respectively. The kappa value for the intraobserver agreement in the identification of the presence of calcification and IPH in carotid plaques was 0.89 and 0.94, respectively. For the interobserver agreement, the kappa value was 0.87 and 0.92 for the identification of the presence of calcification and IPH in carotid plaques, respectively.

## Discussion

This study investigated the changing characteristics of carotid IPH and its potential association with arterial disease risk factors in symptomatic patients. With respect to 45 carotid plaques with IPH, we found that overall volume of carotid IPHs decreased over time with mean annual change of−10.9 mm^3^ (SD: 49.1 mm^3^) over a mean interval of 16 months between the initial and follow-up MR scan. The annual change in IPH volume in the 15 plaques in the progressed IPH group was mean 27.1 mm^3^ (SD: 36.5 mm^3^, range: 2.5–135.2 mm^3^). The annual change in IPH volume in the 30 plaques in the non-progressed IPH group was mean−30.0 mm^3^ (SD: 43.3 mm^3^, range:−138.2–1.78 mm^3^). A total of 22% of plaques exhibited minimal discernible visible change in IPH volume (the annual change in IPH volume >10 mm^3^) in IPH volume, and this includes the 11% with new areas of hemorrhage in the same plaque.

Compared to the results of this study, a much higher prevalence of plaques that overall progressed in IPH volume and had new locations of IPH in the same plaque had been reported (11). Takaya et al. (11) reported that 43% of plaques with IPH had progressed to new plaque hemorrhage in new locations within the carotid plaques after an 18-month follow-up, and the overall change in IPH volume was increased with an average volume change of 34.9 mm^3^, which included the increase in original bleeding areas and new IPH in new locations within the carotid plaques. The authors measured the area of the lumen, outer wall, and tissue components (lipid-rich necrotic core, calcium, and hemorrhage) using a custom-designed imaging analysis tool (QVAS), and the volume of wall and components was calculated. In contrast, in this study, only 5 carotid plaques (11.1%) were observed to have new development of IPHs at other locations of the same plaques and most IPHs increased in size because of the increase in original bleeding areas.

The differences in the results across studies of IPH may be explained by the differences in the study methodology, for example, differences in patient selection criteria (with differences in patients risk factor profiles) and standards of medical intervention (lifestyle interventions and medication) (17–19). Our results support the importance of patient risk factor profiles and medical intervention on IPH progression. In addition, different follow-up times between the studies might be another reason for any inconsistency. However, no differences in the prevalence of IPH progression between the carotid plaques with short follow-up interval and those with longer follow-up time were found in this study.

It has been reported that the hyperintense signal of IPH on T1-weighted sequence was more durable compared with hemorrhage in brain parenchyma (20). The high signal intensity of IPH could last for as long as 54 months without changing to low signal intensity (13). In contrast, the hyperintense signal of parenchymal hemorrhage in brain would mostly decrease within 1 month on T1-weighted images (21). The authors thought that the continuous extravasate of erythrocytes and unique intraplaque environment might contribute to the persistence of IPH (22). Although overall mean volume of carotid IPHs decreased in this study, the hyperintense signal on T1-weighted images in carotid plaques also exhibited significant persistence. None of the 45 plaques with baseline IPH has complete the resolution of the IPH on the follow-up MRI. However, whether the regression of IPH indicates that the plaques were changing to stable or less vulnerable state remains unclear, and further investigations are warranted.

Our results showed that taking an antiplatelet agent at baseline, a baseline history of current or past smoking and larger baseline carotid plaque-containing vessel wall volume were independently associated with plaques in the progressed IPH group. Furthermore, the three independent predictors of being in the IPH progression group were additive, as indicated by the ROC curve analyses. Our findings indicate that awareness and management of such risk factors may be beneficial in lowering the risk of IPH progression in symptomatic patients with carotid atherosclerosis and may also reduce the risk of arterial disease complications, including stroke.

Previous studies investigated the risk factors for the presence of IPH and found that that increasing patient's age, male sex, taking an antiplatelet agent, increase in degree of stenosis, or elevated lipids were the risk factors (10, 19, 23, 24). Of these risk factors, in this study, taking an antiplatelet agent and larger baseline carotid plaque-containing vessel wall volume (a related parameter to increasing degree of stenosis) were also found to be associated with plaques in the progressed IPH group. However, in this study, we did not find that patient's age, male sex, or elevated lipids were predictive of the progressed IPH group. Differences in study findings with respect to the occurrence and progression of IPH may be caused by methodological differences and/or different pathological mechanisms. For example, the occurrence of IPH is thought to be originated from the rupture of immature neovascularization in plaques. However, the progression of IPH is considered to be resulted from increasing bleeding or new vessel rupture in plaques. Furthermore, the arterial disease risk factors for the occurrence and the progression of IPH might be different.

Taking an antiplatelet agent at baseline was reported to be associated with the baseline presence of IPH (19, 24). Mujaj et al. (19) found that patients with carotid IPH were significantly more likely to have used antiplatelet drugs. Furthermore, Liem et al. (24) reported that there was a significant correlation between taking an antiplatelet agent at baseline and carotid IPH in patients with mild to moderate carotid stenosis. In this study, we found that taking an antiplatelet agent at baseline was also predictive of plaques being progressed IPH group. Antiplatelet drugs have antiplatelet effect, which can accelerate the leakage of blood in neotropic vessels and increase IPH volume (25). Our results indicate that the practice around the starting and continuing antiplatelet agents in both asymptomatic and symptomatic patients should be carefully reviewed, given the lack of clear evidence that completely asymptomatic patients now benefit from antiplatelet therapy (26) or that patients with stroke or TIA benefit long-term from antiplatelet therapy and because antiplatelet therapy may increase the risk of stroke or TIA through increasing the risk of carotid intraplaque hemorrhage (27).

Our results showed that the baseline history of current or previous smoking was predictive of plaques being progressed IPH group. Possible mechanism is that the nicotine in cigarettes could accelerate the heart rate and elevate the blood pressure in smokers, which would promote the development of IPH in carotid arteries (28). Previous study (29) has shown that current or previous smoking is the risk factor of cardiovascular events in patients with atherosclerosis. Our results may provide an insight on investigating the mechanism of cardiovascular diseases caused by smoking in patients with atherosclerosis.

Previous studies (11) have shown that the presence of IPH means a greater likelihood of progression in total carotid plaque-containing vessel wall volume compared with those without IPH. In this study, we found that plaques with greater carotid plaque-containing vessel wall volume at baseline also had more rapidly enlarging IPH. Therefore, the changes in IPH volume and total plaque-containing vessel wall volume may promote each other. Larger plaques are more likely to have more complex neovessels and more active inflammation status in them, which could promote an increase in IPH.

Several limitations should be noted in this study. First, the sample size is limited, and the detailed information of taking antiplatelet agent and current or previous history of smoking is missing in this study. Previous studies have shown that the higher dosage antiplatelet agent was also associated with a higher frequency of IPH (19), and longer duration of smoking (30) could increase the risk of stroke in patients with carotid atherosclerosis. Thus, larger studies would be useful to examine the effect of dose of antiplatelet agent and duration of smoking and validate the findings from other researchers.

Second, due to relatively short interval time of follow-up, most of hyperintense signal of IPH on T1-weighted sequence remained unchanged. Therefore, investigations on longer follow-up are required to assess the changing characteristics of signal intensity of IPH and their related influence factors. Third, in this study, all the IPHs were identified on the T1-weighted and TOF images. However, new three dimensional techniques such as magnetization-prepared rapid acquisition gradient echo (MP-RAGE) imaging which exhibited a higher accuracy in the identification and measurement of IPH (31, 32) were not applied. Nevertheless, high sensitivity and specificity (82 and 91%) were still reported in the detection of pathologically confirmed IPH on the T1-weighted and TOF images with positive likelihood ratios of 10.27 and negative likelihood ratios of 0.15 (7, 8, 33). Furthermore, MR imaging has higher diagnostic accuracy for the detection of carotid IPH and higher prediction ability of future arterial disease complications compared with CT angiography (34, 35).

In conclusion, taking an antiplatelet agent at baseline, a baseline history of current or past smoking and larger baseline carotid plaque-containing vessel wall volume were the independent risk factors for IPH progression using the tertile classification method. Our findings indicate that awareness and management of such risk factors may reduce the risk of intraplaque hemorrhage progression and, therefore, may reduce the risk of arterial disease complications, including stroke.

## Data Availability Statement

The original contributions presented in the study are included in the article/supplementary material, further inquiries can be directed to the corresponding authors.

## Ethics Statement

The studies involving human participants were reviewed and approved by Chinese PLA General Hospital. The patients/participants provided their written informed consent to participate in this study.

## Author Contributions

CJ, ZX, and HY contributed to the study conception and design. YF, PP, LS, and ZH collected the data and perform the analysis. LS provided technology support for the image analysis. The first draft of the manuscript was written by LM and ZL. All authors read and approved the final manuscript.

## Conflict of Interest

The authors declare that the research was conducted in the absence of any commercial or financial relationships that could be construed as a potential conflict of interest.

## Publisher's Note

All claims expressed in this article are solely those of the authors and do not necessarily represent those of their affiliated organizations, or those of the publisher, the editors and the reviewers. Any product that may be evaluated in this article, or claim that may be made by its manufacturer, is not guaranteed or endorsed by the publisher.

## Abbreviations

IPH: intraplaque hemorrhage
MRI: magnetic resonance imaging
BMI: body mass index
TOF: time of flight
FOV: field of view
FCR: fibrous cap rupture
ICC: intraclass correlation coefficient
AUC: area under the curve
MP-RAGE: magnetization-prepared rapid acquisition gradient echo
LDL: low-density lipoprotein
HDL: high-density lipoprotein.
